# Walker-Warburg syndrome

**DOI:** 10.1186/1750-1172-1-29

**Published:** 2006-08-03

**Authors:** Jiri Vajsar, Harry Schachter

**Affiliations:** 1 Division of Child Neurology, The Hospital for Sick Children and University of Toronto, 555 University Avenue, Toronto, ON, Canada; 2 Program in Structural Biology and Biochemistry, The Hospital for Sick Children and University of Toronto, 555 University Avenue, Toronto, ON, Canada

## Abstract

Walker-Warburg Syndrome (WWS) is a rare form of autosomal recessive congenital muscular dystrophy associated with brain and eye abnormalities. WWS has a worldwide distribution. The overall incidence is unknown but a survey in North-eastern Italy has reported an incidence rate of 1.2 per 100,000 live births. It is the most severe form of congenital muscular dystrophy with most children dying before the age of three years. WWS presents at birth with generalized hypotonia, muscle weakness, developmental delay with mental retardation and occasional seizures. It is associated with type II cobblestone lissencephaly, hydrocephalus, cerebellar malformations, eye abnormalities and congenital muscular dystrophy characterized by hypoglycosylation of α-dystroglycan. Several genes have been implicated in the etiology of WWS, and others are as yet unknown. Several mutations were found in the Protein O-Mannosyltransferase 1 and 2 (*POMT1 and POMT2*) genes, and one mutation was found in each of the fukutin and fukutin-related protein (*FKRP*) genes. Laboratory investigations usually show elevated creatine kinase, myopathic/dystrophic muscle pathology and altered α-dystroglycan. Antenatal diagnosis is possible in families with known mutations. Prenatal ultrasound may be helpful for diagnosis in families where the molecular defect is unknown. No specific treatment is available. Management is only supportive and preventive.

## Disease name/synonyms

Walker-Warburg syndrome

Cerebroocular dysgenesis (COD)

Cerebroocular dysplasia-muscular dystrophy (COD-MD) syndrome

Chemke syndrome

Hydrocephalus-Agyria-Retinal Dysplasia (HARD) Syndrome

HARD +/- syndrome

Pagon syndrome

Warburg syndrome

## Definition

Walker-Warburg Syndrome (WWS) is a genetically heterogeneous disease presenting with congenital muscular dystrophy, type II lissencephaly, hydrocephalus, cerebellar malformations and eye abnormalities [1-4]. So far, only 10%–20% of cases can be confirmed by DNA analysis of mutations in the Protein O-Mannosyltransferase 1 (*POMT1*) gene [5-7]. DNA analysis in other genes besides *POMT1 *can be confirmative: *fukutin *[8,9]; *FKRP *[10] and *POMT2 *[11].

## Epidemiology

WWS is a rare disease with a worldwide distribution; however, the overall incidence is unknown. A survey in north-eastern Italy has reported an incidence rate of 1.2 per 100,000 live births [12].

## Clinical description

WWS is the most severe congenital muscular dystrophy (CMD). Symptoms and signs are already present at birth and early infancy, and occasionally can be detected prenatally with imaging techniques. WWS is associated with generalized hypotonia, muscle weakness, developmental delay with mental retardation and, in some children, seizures. There may be a variety of anterior eye anomalies (cataracts, shallow anterior chamber, microcornea and microphthalmia, and lens defects) and a spectrum of posterior eye anomalies (retinal detachment or dysplasia, hypoplasia or atrophy of the optic nerve and macula and coloboma). Glaucoma or buphthalmos may be present. Brain abnormalities include migrational defect with type II lissencephaly (cobblestone type), hydrocephalus, vermal or general cerebellar hypoplasia and flat brainstem with small pyramids. White matter shows hypomyelination. Additional brain anomalies such as hypoplasia/agenesis of corpus callosum, occipital encephalocele and Dandy-Walker malformation have been described. Other recognized associated anomalies are small penis, undescended testes, and, rarely, other facial dysmorphic features such as low set or prominent ears and cleft lip or palate.

Laboratory investigations usually show elevated creatine kinase, myopathic/dystrophic muscle pathology and altered α-dystroglycan.

## Etiology

The dystrophin glycoprotein complex (DGC) is an assembly of proteins spanning the sarcolemma of skeletal muscle cells [13-15]. Defects in the DGC appear to play critical roles in several muscular dystrophies due to disruption of basement membrane organization [16,17]. Dystroglycan (dystrophin-associated glycoprotein), a major component of the DGC, is expressed in many cell types, and is synthesized as a precursor propeptide that is post-translationally cleaved and differentially glycosylated to yield α- and β-dystroglycans. There is evidence suggesting that the tetrasaccharide NeuAcα2, 3Galβ1, 4GlcNAcβ1, 2Manα1-O-Ser/Thr present on α-dystroglycan is required for the binding of laminin and other ligands to α-dystroglycan [18-20].

Mutations interfering with the binding of extracellular ligands to α-dystroglycan may lead to weakened anchorage of muscle fibers to the extracellular matrix with very early (*i.e*., embryonic) and rapid muscle dysfunction and necrosis [17]. This process is responsible for an etiologically heterogeneous group of autosomal recessive CMDs with associated brain and eye abnormalities [5,21-28]. Immunohistochemical studies using antibodies against α-dystroglycan (*e.g*. VIA4-1 directed against the glycans on α-dystroglycan) have revealed that these CMDs are associated with deficient immunostaining of α-dystroglycan in the basal lamina of skeletal muscles [29-33]. The data indicate that α-dystroglycan is underglycosylated in muscle from these patients [34].

A genome-wide linkage analysis in 10 consanguineous families with WWS indicated the existence of at least three WWS loci [6]. Since protein O-mannoside β1,2-N-acetylglucosaminyltransferase (POMGnT1) had recently been implicated as the cause of muscle-eye-brain disease (MEB) [35-37], Beltran-Valero de Bernabe *et al*. [6] screened WWS patients for mutations in the gene encoding protein O-mannosyltransferase (POMT) that attaches mannose *via *a O-glycosidic link to the Ser/Thr residues of α-dystroglycan, thereby synthesizing the substrate for POMGnT1. Homozygous mutations at the *POMT1 *locus on chromosome 9q34 were found in 5 out of 15 consanguineous families with WWS. Sequencing of the *POMT1 *gene revealed mutations in 6 out of 30 unrelated patients with WWS [6]. Of the five mutations identified, two were nonsense mutations, two were frameshift mutations and one was a missense mutation. Other mutations and phenotype-genotype correlations were reported in 2006 [38]. More severe phenotypes were associated with highly disruptive homozygous mutations, while mild compound heterozygous mutations in one or both alleles were associated with a less severe phenotype and longer life span.

In another series of 30 patients with classic WWS, two potential novel heterozygous mutations in the *POMT1 *gene were found in two patients from non-consanguineous parents [39]. However, the other 28 patients failed to show any *POMT1 *mutations. Linkage analysis in six consanguineous families with WWS showed that defective *POMT1 *was an uncommon cause of WWS in this population (the incidence of coding region mutations was less than 7%).

One other child with the WWS phenotype was reported to have a homozygous null mutation in the *fukutin *gene and another child a homozygous C953T missense mutation in the *FKRP *gene [8,10]. A fourth causative gene for WWS was found in 2005, when three homozygous mutations in the *POMT2 *gene were detected in WWS patients from three families [7].

In all tissues tested, *POMT1 *mRNA is expressed as a 3.1 kb transcript, with highest levels in the testes and fetal brain. Alternative splicing of several exons in all tissues predicts the generation of several protein isoforms. A method for measuring POMT activity in mammalian cells has been developed [37]. However, recombinant POMT1 was found to be inactive. It turns out that the human genome contains two genes encoding POMT (*POMT1 *and *POMT2*). Neither recombinant protein by itself shows enzyme activity. Mixing of recombinant forms of the two proteins also fails to result in an active enzyme. The two genes must be co-expressed to obtain an active POMT enzyme complex [40-42].

Homozygous inactivation of the *POMT1 *gene in the mouse is embryonic lethal with developmental arrest around embryonic day (E) 7.5 and death between E7.5 and E9.5 [43]. The *POMT1*(-/-) embryos present defects in the formation of Reichert's membrane, the first basement membrane to form in the embryo. The failure of this membrane to form appears to be the result of abnormal glycosylation and maturation of dystroglycan that may impair recruitment of laminin, a structural component required for the formation of Reichert's membrane in rodents.

## Diagnostic methods and criteria

The diagnosis is established on the basis of four criteria:

• congenital muscular dystrophy characterized by hypoglycosylation of α-dystroglycan

• high creatine kinase level

• anterior or posterior eye anomalies

• migrational brain defect with type II lissencephaly and hydrocephalus

• abnormal brainstem/cerebellum

Mutations or gene loci are currently unknown in majority of patients. Only 10 – 20% of cases have a mutation in the *POMT1*, *POMT2*, *fukutin *or *FKRP *genes.

The diagnostic criteria for WWS were established on the basis of data obtained from 21 patients and an additional 42 patients from the literature [5]. Four abnormalities were present in all patients checked for these anomalies: type II lissencephaly (21/21), cerebellar malformation (20/20), retinal malformation (18/18), and CMD (14/14). Two other frequently observed abnormalities, ventricular dilatation with or without hydrocephalus (20/21) and anterior chamber malformation (16/21), were helpful but not obligatory for diagnosis because they were not constant. All other abnormalities occurred less frequently. Congenital macrocephaly with hydrocephalus (11/19) was more common than congenital microcephaly (3/19). Dandy-Walker malformation (10/19) was sometimes associated with posterior cephalocele (5/21). Additional abnormalities included slit-like ventricles (1/21), microphthalmia (8/21), ocular colobomas (3/15), congenital cataracts (7/20), and genital anomalies in males (5/8).

## Differential diagnosis

The differential diagnoses include:

• Fukuyama congenital muscular dystrophy (FCMD)

• CMD type 1C

• CMD type 1D

• Muscle-eye-brain disease (MEB)

• Congenital muscular dystrophies without brain and eye abnormalities

FCMD and MEB are associated with similar but less severe brain and eye changes and CMD. Patients with CMD type 1C may have variable findings on brain magnetic resonance imaging (MRI) and eye exam. The only patient with CMD type 1D reported so far has milder brain abnormalities than those seen in WWS.

### Genetic counseling

WWS is an autosomal recessive disorder. In families with one affected child, the risk of having another child with the disease is 25%.

## Antenatal diagnosis

Antenatal molecular diagnosis might be possible in families with known mutations. Antenatal ultrasound can also be helpful in families in whom the molecular defect is unknown. Hydrocephalus has been detected by ultrasound as early as at 13 weeks of gestation [44] and occipital encephalocele, not always present, has been detected at 18 weeks of gestation [45].

## Management including treatment

Management is only supportive and preventive. If seizures develop, they usually need to be treated with anticonvulsants. A few children require a neurosurgical procedure such as shunting of hydrocephalus or encephalocele operation. Physical therapy can be offered to facilitate development or prevent worsening of contractures although its efficacy has not been established. Feeding needs to be monitored and supplemental nasogastric or gastric tube feeding provided in some cases. Detailed eye assessment is also needed.

## Prognosis

The condition is usually lethal within the first few months of life, with almost all children dying by the age of three.

## Unresolved questions

There are no known candidate genes for 80%–90% of children with WWS. Although the *POMT1 *gene involved in WWS implies a defect in O-mannosylation of α-dystroglycan, the exact pathophysiology of this disorder is not fully understood. As new genetic defects associated with WWS are reported, a better genotype-phenotype correlation should be established.

### Other interesting issues

The following research papers have helped to shed light on Walker-Warburg syndrome:

#### Role of dystroglycan in pathogenesis [46]

In mice, brain-selective deletion of dystroglycan is sufficient to cause CMD-like brain malformations, including disarray of cerebral cortical layering, fusion of cerebral hemispheres and cerebellar folia, and aberrant migration of granule cells. Additionally, high-affinity binding to laminin is lost, and there are discontinuities in the pial surface basal lamina (glia limitans) that probably underlie the neuronal migration errors. Furthermore, mutant mice have severely blunted hippocampal long-term potentiation with electrophysiologic characterization indicating that dystroglycan might have a postsynaptic role in learning and memory.

The data strongly support the hypothesis that defects in dystroglycan are central to the pathogenesis of structural and functional brain abnormalities seen in CMD.

#### Pathogenetic mechanisms underlying degeneration [47]

Immunohistochemical and electron microscopy were carried out on muscle obtained by biopsy from a patient with WWS carrying a homozygous frameshift mutation in *POMT1*. The α-dystroglycan glycosylated epitope was not detected in muscle fibers or intramuscular peripheral nerves. Laminin-α2 chain and perlecan were reduced in muscle fibers but well preserved in intramuscular peripheral nerves. The basal lamina in several muscle fibers showed discontinuities and detachment from the plasmalemma. Most nuclei, including myonuclei and satellite cell nuclei, showed detachment or complete absence of peripheral heterochromatin from the nuclear envelope. Apoptotic changes were detected in 3% of muscle fibers.

The particular combination of basal lamina and nuclear changes suggests that a complex pathogenetic mechanism, involving several subcellular compartments, underlies the muscle degenerative process in WWS.

#### Genetic heterogeneity [48]

Immunohistochemistry and immunoblotting showed almost complete absence of α-dystroglycan and a mild reduction of laminin-α2 in two patients with WWS. In contrast, immunohistochemical labeling of perlecan and collagen VI was normal. Linkage analysis excluded the *POMT1 *locus, confirming that WWS is a genetically heterogeneous condition.

It was suggested that disruption of the α-dystroglycan/laminin-α2 axis in the basal lamina may play a role in the degeneration of muscle fibers in WWS patients who do not have a mutation in *POMT1*.
